# Efficient parameter extraction of photovoltaic models with a novel enhanced prairie dog optimization algorithm

**DOI:** 10.1038/s41598-024-58503-y

**Published:** 2024-04-04

**Authors:** Davut Izci, Serdar Ekinci, Abdelazim G. Hussien

**Affiliations:** 1 Department of Computer Engineering, Batman University, Batman, 72100 Turkey; 2 MEU Research Unit, Middle East University, Amman, Jordan; 3 Department of Computer and Information Science, Linköping University, Linköping, Sweden; 4 Faculty of Science, Fayoum University, Fayoum, Egypt; 5 Applied Science Research Center, Applied Science Private University, Amman, 11931 Jordan

**Keywords:** Prairie dog optimization, Solar energy, Parameter extraction, Logarithmic spiral search, Random learning mechanism, Mathematics and computing, Computer science, Electrical and electronic engineering

## Abstract

The growing demand for solar energy conversion underscores the need for precise parameter extraction methods in photovoltaic (PV) plants. This study focuses on enhancing accuracy in PV system parameter extraction, essential for optimizing PV models under diverse environmental conditions. Utilizing primary PV models (single diode, double diode, and three diode) and PV module models, the research emphasizes the importance of accurate parameter identification. In response to the limitations of existing metaheuristic algorithms, the study introduces the enhanced prairie dog optimizer (En-PDO). This novel algorithm integrates the strengths of the prairie dog optimizer (PDO) with random learning and logarithmic spiral search mechanisms. Evaluation against the PDO, and a comprehensive comparison with eighteen recent algorithms, spanning diverse optimization techniques, highlight En-PDO’s exceptional performance across different solar cell models and CEC2020 functions. Application of En-PDO to single diode, double diode, three diode, and PV module models, using experimental datasets (R.T.C. France silicon and Photowatt-PWP201 solar cells) and CEC2020 test functions, demonstrates its consistent superiority. En-PDO achieves competitive or superior root mean square error values, showcasing its efficacy in accurately modeling the behavior of diverse solar cells and performing optimally on CEC2020 test functions. These findings position En-PDO as a robust and reliable approach for precise parameter estimation in solar cell models, emphasizing its potential and advancements compared to existing algorithms.

## Introduction

Solar energy, a pivotal natural resource with the potential for electricity conversion, has garnered increasing attention within the realm of renewable energy sources^1^. To harness solar energy effectively, intricate conversion processes are essential to meet the escalating energy demands of contemporary society^2^. Photovoltaic (PV) plants, functioning as crucial instruments in this transformation, face challenges due to exposure to severe weather conditions. While these outdoor installations efficiently convert the sun's radiant energy into electrical power, their performance is susceptible to environmental factors, necessitating precise parameter extraction methods^3^.

The accuracy of PV system parameter extraction is a paramount research focus, given the complexities arising from real-world operational conditions, aging effects, and the absence of instrumentation in practical settings. Achieving precise identification of PV system parameters is particularly critical for enhancing the efficiency of PV models under diverse environmental conditions. The primary PV model systems, namely the single diode (SD) and double diode (DD) models, are widely employed^4^, alongside advanced representations like the three diode (TD) model, offering a more accurate description of PV cell behavior^5^. Additionally, PV module models have been adopted for parameter extraction^6,7^. Augmenting the accuracy of parameter identification for these solar cell models is of utmost importance.

Numerous methodologies, including analytical methods^8^, numerical operations^9^, and metaheuristic algorithms^10^, have been developed for PV cell parameter identification. Analytical methods, while providing quick solutions, often sacrifice accuracy due to approximations. Numerical methods, dependent on random initial value selection, face limitations as the number of identification parameters increases. In recent years, metaheuristic optimization algorithms have emerged as superior alternatives, offering simplicity, fewer restrictive conditions, and robustness^11–14^. Inspired by natural behavior, a wide range of metaheuristic and heuristic methods have proven effective in identifying photovoltaic cell parameters.

While metaheuristic algorithms have been used successfully for PV parameter estimation, their exploration and exploitation limitations have prompted the development of improved metaheuristic algorithms. Several enhanced metaheuristic algorithms have been specifically developed for identifying parameters in PV systems. For instance, in^15^ the authors proposed a hybrid algorithm that combines teaching–learning-based optimization and artificial bee colony to improve the accuracy and reliability of PV parameter estimation. In another work, the authors proposed a multiple learning backtracking search algorithm that combines population diversity and exploration ability through simultaneous learning from current and historical population information^16^. In^17^, the development of an accurate model for PV systems was performed through the application of the evaporation rate-based water cycle algorithm for parameter estimation. An improved ant lion optimizer that incorporates chaotic sequence initialization, particle swarm algorithm-inspired position update, and dynamic contraction regions was also developed as an efficient tool for parameter identification of photovoltaic cells^18^. A hybrid approach combining an improved adaptive Nelder-Mead simplex algorithm with artificial bee colony algorithm using a new eagle strategy was also proposed in the literature^19^. It is feasible to list more examples that have been developed for parameter identification in PV systems.

In 2022, Ezugwu et al. introduced the prairie dog optimizer (PDO), a nature-inspired metaheuristic algorithm inspired by prairie dogs' foraging and burrow-building behaviors^20^ and has been demonstrated to be an effective optimizer for engineering problems^21^. However, PDO exhibits limitations, including a tendency to get trapped in local best solutions and lower convergence accuracy. To address these issues, random learning^22,23^ and logarithmic spiral search^24,25^ mechanisms were integrated into PDO, resulting in the enhanced PDO (En-PDO). Evaluation on CEC2020 test functions consistently demonstrated En-PDO's superiority over PDO, marked by lower mean values and significant p-values.

En-PDO, designed for accurate estimation of PV model parameters, integrates PDO’s strengths with random learning and logarithmic spiral search mechanisms, aiming for superior global and local search capabilities. The algorithm's efficacy was tested on SD, DD, TD models, and the PV module model. En-PDO consistently outperformed PDO, showcasing superior convergence behavior and lower error values. Moreover, a comprehensive comparison with eighteen recent algorithms (improved moth flame algorithm with local escape operators^26^, ranking teaching–learning-based optimization^27^, dynamic leader multi-verse optimizer^28^ amended reptile search algorithm^29^, chaos game optimization-least squares algorithm^30^, artificial hummingbird optimization^31^, elite learning adaptive differential evolution^32^, squirrel search algorithm^33^, enhanced gradient-based optimizer^34^, random reselection particle swarm optimization^35^, sine cosine differential gradient based optimizer^36^, differential evolution algorithm^37^, tree seed algorithm^38^, Manta ray foraging optimization^39^, bald eagle search algorithm^40^, stochastic fractal search algorithm^41^, coyote optimization algorithm^42^ and slime mould algorithm^43^), spanning diverse optimization techniques, reaffirmed En-PDO's exceptional performance across different solar cell models.

To assess the effectiveness of En-PDO, it is applied to estimate parameters for the SD, DD, TD, and PV module models using standard experimental datasets (R.T.C. France silicon solar cell^33^ for SD, DD and TD models and Photowatt-PWP201 solar cell^44^ for PV module model). The comparison results clearly highlight the superior performance of the En-PDO as it consistently achieves competitive or superior performance in terms of root mean square error values across all models. Notable achievements include the lowest root mean square values in the SD, DD, TD, and PV models, demonstrating the efficacy of En-PDO in accurately modeling the behavior of different solar cells. These findings underscore the efficacy and potential of the En-PDO as a robust and reliable approach for parameter estimation in solar cell models.

## Structure of enhanced prairie dog optimizer

### Overview of prairie dog optimizer

The Prairie dog optimizer (PDO), introduced in 2022, simulates prairie dogs' foraging and burrow-building behaviors for optimization purposes^20^. It begins with a random search pattern to identify the region of interest and emphasizes exploration and exploitation during the updating process. The optimization process involves three mathematical phases, namely initialization and evaluation, exploration and exploitation. PDO initializes a colony of $Q$ coteries, each containing $N$ prairie dogs with position vectors. The colony is represented by a matrix $C$, and each coterie by a matrix $PA$. Initialization is done using uniform distribution.

1 $$C_{ij} = P_{j}^{Lb} + U\left( {0,1} \right).\left( {P_{j}^{Ub} - P_{j}^{Lb} } \right)$$

2 $$P_{ij} = P_{j}^{lb} + U\left( {0,1} \right).\left( {P_{j}^{ub} - P_{j}^{ub} } \right)$$

Fitness evaluation is based on the objective function, aiming for the minimum fitness value within the colony. Prairie dogs explore (exploration phase) using Levy flight and digging strength to find new solutions. The position updates are modeled as follows:

3 $$P_{i + 1,j + 1} = P_{best,j} + C_{best,i,j} \times \rho - Z_{i,j} \times Levy\left( n \right)\;\;\forall t < T_{max} /4$$

4 $$P_{i + 1,j + 1} = P_{best,j} + P_{r,j} \times DS \times Levy\left( n \right)\;\;\forall T_{max} /4 \le t < T_{max} /2$$

where $\rho$, $C_{best,i,j}$, $P_{r,j}$, $Z_{i,j}$, $Ds$ and $Levy\left( n \right)$ are the food source alert parameter, the effects of the most effective solution, a randomly created solution, the random cumulative influence of each prairie dog inside the colony, the coterie's strength in digging and Levy distribution function^20^.

**Figure 1:**
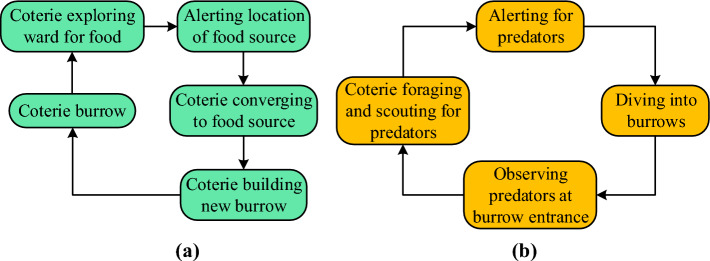
Model of the exploration (**a**) and exploitation phases (**b**).

The exploitation phase involves simulating prairie dogs' communication skills. Position updates are modeled as:

5 $$P_{i + 1,j + 1} = P_{best,j} - C_{best,i,j} \times \varepsilon - Z_{i,j} \times rand\;\;\forall T_{max} /2 \le iter < 3T_{max} /4$$

6 $$P_{i + 1,j + 1} = P_{best,j} + Pe \times rand\;\;\forall 3T_{max} /4 \le iter < T_{max}$$

where $\varepsilon$, $Pe$ and $rand$ are a small number to illustrate the goodness of food source, the effects of the predator and a random value between $0$ and $1$ generated by the uniform distribution, respectively. The PDO framework (model of the exploration and exploitation phases) is represented in Fig. 1.

### Proposed enhanced prairie dog optimizer

**Figure 2:**
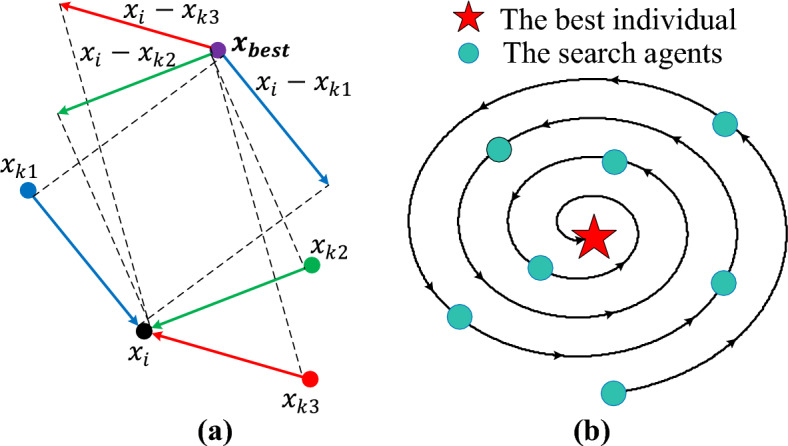
Searching principles of random learning (**a**) and logarithmic spiral search (**b**) mechanisms.

This study proposes an enhanced version of the PDO (En-PDO) by incorporating the random learning (RL) and logarithmic spiral search (LSS) mechanisms into the original PDO. The RL mechanism is a machine learning technique used to improve the exploration capacity^45^. The RL mechanism can be described as $x_{i}^{RL} \left( t \right) = x_{best} \left( t \right) + rand \times \left( {x_{i} \left( t \right) - x_{k1} \left( t \right)} \right)$ for $f_{k1} < f_{k2}\, {\text{and}}\, f_{k1} < f_{k3}$; $x_{i}^{RL} \left( t \right) = x_{best} \left( t \right) + rand \times \left( {x_{i} \left( t \right) - x_{k2} \left( t \right)} \right)$ for $f_{k2} < f_{k1} \,{\text{and}}\, f_{k2} < f_{k3}$; $x_{i}^{RL} \left( t \right) = x_{best} \left( t \right) + rand \times \left( {x_{i} \left( t \right) - x_{k3} \left( t \right)} \right)$ for the rest of the relationships between fitness functions ($f_{k1}$, $f_{k2}$ and $f_{k3}$) of random individuals ($x_{k1} \left( t \right)$, $x_{k2} \left( t \right)$ and $x_{k3} \left( t \right)$) by assuming $x_{best}$ as the optimal solution and $x_{i} \left( t \right)$ as the $i^{th}$ individual in the $t^{th}$ iteration’s population. This study also adopts LSS mechanism^46^ which can be expressed with $x_{i}^{Ls} \left( t \right) = \left| {x_{best} \left( t \right) - x_{i} \left( t \right)} \right| \cdot e^{\alpha l} \cdot \cos \left( {2\pi l} \right) + x_{best} \left( t \right)$ where $l$ is a random variable within the range $\left[ { - 1,1} \right]$, calculated as $l = 2 \times rand - 1$, $\alpha$ is a constant set to $1$, shaping the spiral, and $x_{best} \left( t \right)$ represents the optimal position in the current iteration. Figure 2 demonstrates the searching principles of the RL and LSS mechanisms employed in this study.

The proposed En-PDO algorithm incorporates a selective structure, as well, for further enhancing the performance. As part of the latter approach, the position update is performed as follows. The current solution, $x_{i} \left( t \right)$, is replaced by the newly obtained solution, $x_{i}^{Ls} \left( t \right)$, in the event that $x_{i} \left( t \right)$ exhibits equal or superior fitness. Otherwise, $x_{i} \left( t \right)$ remains within the population. This selection mechanism effectively prevents the retention of suboptimal solutions. In essence, superior new solutions are continually refined over successive iterations, while inferior ones are systematically discarded.

**Figure 3:**
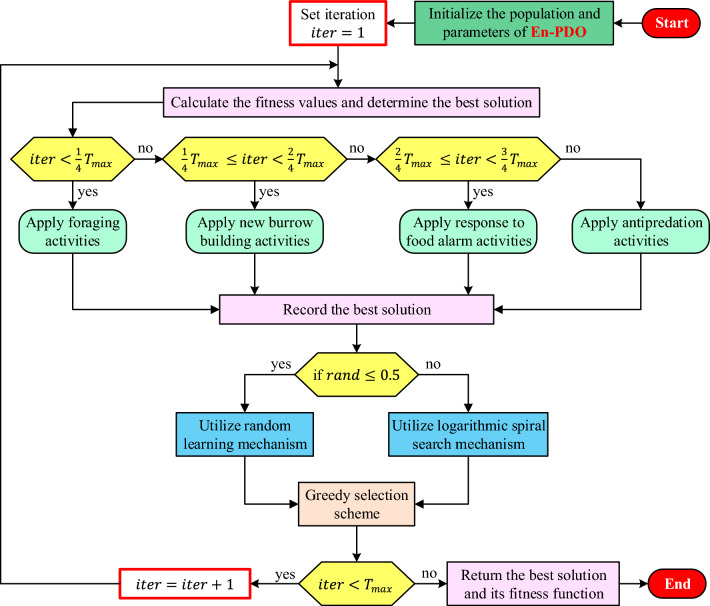
Flowchart of En-PDO algorithm.

Figure 3 provides a detailed flowchart of the proposed En-PDO algorithm. As can be observed from this flowchart, the En-PDO starts with the original PDO then the best solution is further processed using RL or LSS mechanisms. The adoption of the latter two mechanisms is decided randomly by providing equal chances two those mechanisms. In this way, an efficient structure that can be used for the parameter extraction of the PV models is achieved.

## Performance assessment of proposed En-PDO on CEC2020 test functions

To evaluate the efficacy of the proposed En-PDO, a comprehensive analysis was conducted using the CEC2020 benchmark test functions. The experimental setup involved 30 independent runs with a population size of 50 and a total of 1000 iterations for each algorithm. Table 1 provides a summary of the CEC2020 test functions utilized in the assessment. These functions vary in type, name, lower and upper bounds, and the global optimum. The En-PDO algorithm was tested against the PDO on these diverse functions.

**Table 1:** Descriptions of CEC2020 test functions.

Function	Type of function	Name	Lower bound	Upper bound	Global
$F_{CEC2020 - 01}$	Unimodal	Shifted and rotated bent cigar function	− 100	100	100
$F_{CEC2020 - 02}$	Basic	Shifted and rotated Schwefel’s function	− 100	100	1100
$F_{CEC2020 - 03}$		Shifted and rotated Lunacek bi-Rastrigin function	− 100	100	700
$F_{CEC2020 - 04}$		Expanded Rosenbrock’s plus Griewank’s function	− 100	100	1900
$F_{CEC2020 - 05}$	Hybrid	Hybrid function 1 ($N = 3$)	− 100	100	1700
$F_{CEC2020 - 06}$		Hybrid function 2 ($N = 4$)	− 100	100	1600
$F_{CEC2020 - 07}$		Hybrid function 3 ($N = 5$)	− 100	100	2100
$F_{CEC2020 - 08}$	Composition	Composition function 1 ($N = 3$)	− 100	100	2200
$F_{CEC2020 - 09}$		Composition function 2 ($N = 4$)	− 100	100	2400
$F_{CEC2020 - 10}$		Composition function 3 ($N=5$)	− 100	100	2500

Table 2 presents the statistical performance evaluation for unimodal and basic function types. The results highlight the minimum, maximum, mean, standard deviation, and the p-value from Wilcoxon's signed-rank test for both En-PDO and PDO on each function. Notably, the En-PDO consistently outperformed PDO, evident in its lower mean and significant p-values, asserting its superiority.

**Table 2:** Statistical performance evaluation of unimodal and basic function types.

Function	Algorithm	Minimum	Maximum	Mean	Standard deviation	p-value
$F_{CEC2020 - 01}$	En-PDO	100	100	100	9.5359E − 13	1.7344E−06 (Winner: En-PDO)
	PDO	104.2	4125.7	1456.6	1118.4	
$F_{CEC2020 - 02}$	En-PDO	1100.1	1123.4	1104.2	5.4881	1.7344E−06 (Winner: En-PDO)
	PDO	1195.6	1758.5	1449.5	156.59	
$F_{CEC2020 - 03}$	En-PDO	700	706.29	703.73	2.1169	1.7344E−06 (Winner: En-PDO)
	PDO	713.08	738.93	725.28	5.7414	
$F_{CEC2020 - 04}$	En-PDO	1900	1900	1900	0	0.5 (Winner: Tie)
	PDO	1900	1900	1900	0.0056426	

Table 3 extends the evaluation to hybrid and composition function types. En-PDO's performance is once again evident, demonstrating lower mean values, smaller standard deviations, and consistently significant p-values compared to PDO. The statistical outcomes affirm the superior performance of En-PDO across a diverse range of CEC2020 functions.

**Table 3:** Statistical performance evaluation of hybrid and composition function types.

Function	Algorithm	Minimum	Maximum	Mean	Standard deviation	p-value
$F_{CEC2020 - 05}$	En-PDO	1710.3	1751.1	1723	9.0279	1.7344E−06 (Winner: En-PDO)
	PDO	1803.7	20,078	4955.6	3888.9	
$F_{CEC2020 - 06}$	En-PDO	1600.3	1601.3	1600.9	0.27535	1.7333E−06 (Winner: En-PDO)
	PDO	1601.4	1641.7	1615.5	12.228	
$F_{CEC2020 - 07}$	En-PDO	2100.8	2103.6	2101.6	0.69349	1.7344E−06 (Winner: En-PDO)
	PDO	2429.1	14,289	4718.8	2846.7	
$F_{CEC2020 - 08}$	En-PDO	2200	2301.5	2249.6	48.956	0.00066392 (Winner: En-PDO)
	PDO	2221.1	2367.6	2293.2	44.178	
$F_{CEC2020 - 09}$	En-PDO	2400	2739.5	2525	105.7	0.036821 (Winner: En-PDO)
	PDO	2516.1	2751.6	2562.2	67.058	
$F_{CEC2020 - 10}$	En-PDO	2500	2847.4	2727.4	116.92	1.7344E−06 (Winner: En-PDO)
	PDO	2853.7	2899	2870.5	13.261	

The consistent dominance of En-PDO across various function types underscores its efficacy in achieving competitive and reliable optimization results. The algorithm’s ability to navigate both unimodal and more complex hybrid and composition functions positions En-PDO as a robust optimization tool. These findings showcase the potential of En-PDO for addressing optimization challenges across different problem domains, making it a promising choice for practical applications.

## Problem formulation of solar photovoltaic system

### Single-diode model

**Figure 4:**
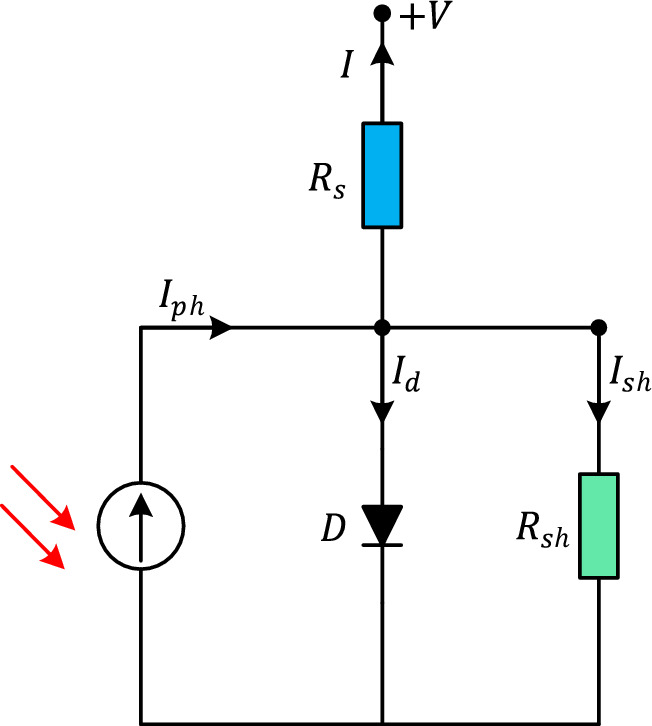
Equivalent circuit of SD model.

The single-diode (SD) model offers a simplified mathematical representation of the electrical characteristics exhibited by a PV cell. Despite its simplicity, the SD model manages to capture the essential aspects of the PV cell's electrical response while providing a computationally efficient representation. In the SD model, the current–voltage (I-V) relationship of a PV cell is defined by the following equation:

7 $$I = I_{ph} - I_{sd} \left[ {e^{{\frac{{\left( {V + IR_{s} } \right)}}{{\left( {nV_{t} } \right)}}}} - 1} \right] - \frac{{\left( {V + IR_{s} } \right)}}{{R_{sh} }}$$

where $I$ is the output current of the PV cell, $V$ is the voltage across the PV cell terminals, $I_{ph}$ is the photocurrent generated by the cell under illumination, $I_{sd}$ is the diode saturation current, $R_{s}$ is the series resistance of the cell, $R_{sh}$ is the shunt resistance of the cell, $n$ is the diode ideality factor, $V_{t}$ is the thermal voltage, approximately equal to $kT/q$, where $k$ is Boltzmann's constant, $T$ is the temperature in Kelvin, and $q$ is the elementary charge. Figure 4 illustrates the conceptual depiction of a solar PV cell employing the single-diode model.

### Double-diode model

**Figure 5:**
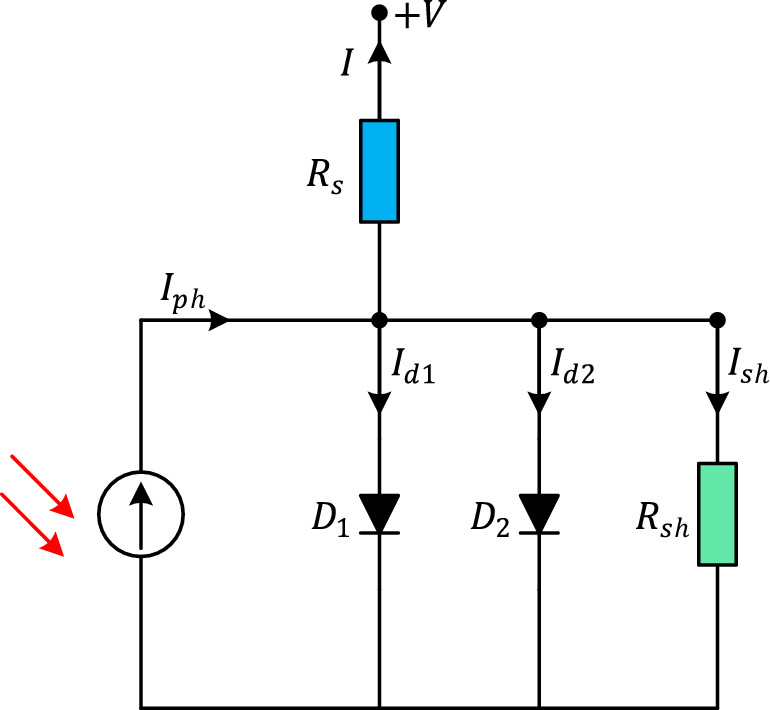
Equivalent circuit of DD model.

The double-diode (DD) model represents an advanced approach to PV cell modeling that incorporates additional diodes to capture more complex electrical behavior. In the DD model, the current–voltage (I–V) relationship of a PV cell is defined by the following equation:

8 $$I = I_{ph} - I_{sd1} \left[ {e^{{\frac{{\left( {V + IR_{s} } \right)}}{{\left( {n_{1} V_{t} } \right)}}}} - 1\left] { - I_{sd2} } \right[e^{{\frac{{\left( {V + IR_{s} } \right)}}{{\left( {n_{2} V_{t} } \right)}}}} - 1} \right] - \frac{{\left( {V + IR_{s} } \right)}}{{R_{sh} }}$$

where $I_{sd1}$ is the diode saturation current of the main diode, $I_{sd2}$ is the diode saturation current of the additional diode, $n_{1}$ is the ideality factor of the main diode and $n_{2}$ is the ideality factor of the additional diode. Figure 5 illustrates the conceptual depiction of a solar PV cell employing the DD model.

### Three-diode model

**Figure 6:**
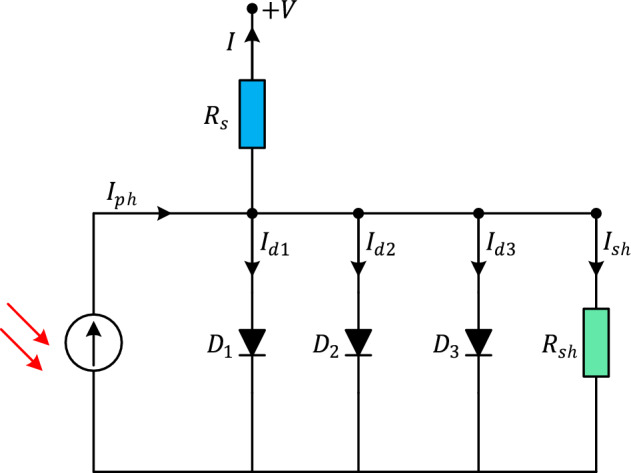
Equivalent circuit of TD model.

The three-diode (TD) model is an advanced representation of a PV cell that provides a more accurate description of its behavior compared to simpler models. In this model, the current–voltage relationship is given as $I = I_{ph} - I_{d1} - I_{d2} - I_{d3} - I_{sh}$ where $I_{d1}$ is the current through the ideal diode; $I_{d2}$ is the current through the recombination diode and $I_{d3}$ is the current through the shunt diode. Considering this explanation, the overall current through the PV cell can be calculated by summing up the currents through the three diodes in TD model:

9 $$I = I_{ph} - I_{sd1} \left( {e^{{\frac{{V + IR_{s} }}{{n_{1} V_{t} }} - 1}} } \right) - I_{sd2} \left( {e^{{\frac{{V + IR_{s} }}{{n_{2} V_{t} }} - 1}} } \right) - I_{sd3} \left( {e^{{\frac{{V + IR_{s} }}{{n_{3} V_{t} }} - 1}} } \right) - \frac{{V + IR_{s} }}{{R_{sh} }}$$

where $n_{1}$, $n_{2}$ and $n_{3}$ are the ideality factors of the diodes $D_{1}$, $D_{2}$ and $D_{3}$, respectively. Figure 6 illustrates the equivalent circuit of a solar PV cell employing the three-diode model.

### Photovoltaic module model

**Figure 7:**
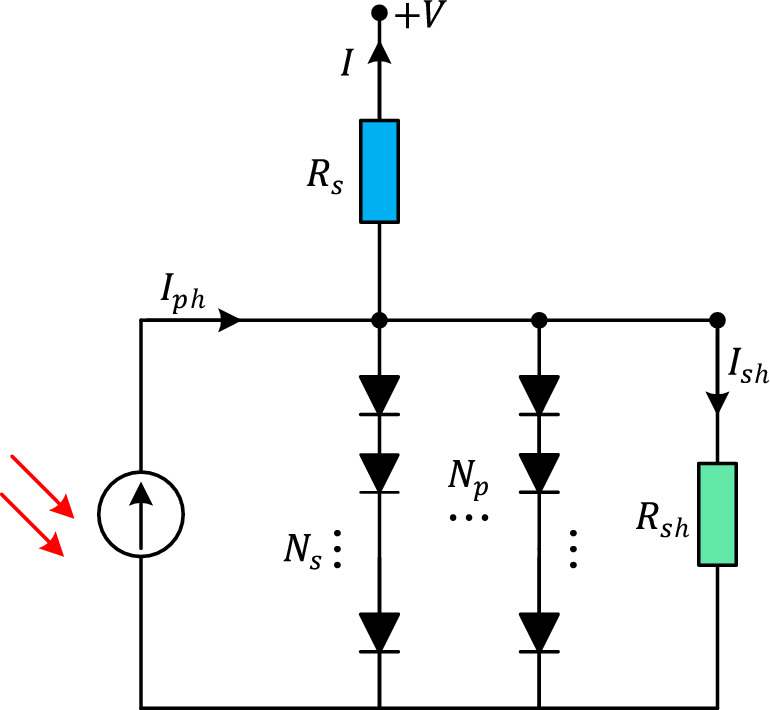
Equivalent circuit of PV module model.

The photovoltaic (PV) module model captures the relationship between the incident solar irradiance, temperature, and the electrical characteristics of the module. The model assumes that the PV module can be represented as a single diode connected in parallel with a current source. Figure 7 represents the equivalent circuit of a PV module where $N_{p}$ and $N_{s}$ are denoting the number of cells in parallel and series respectively.

Since the solar cells are connected in series largely, the $N_{p}$ value equals to 1. Therefore, the mathematical model of a PV module can be represented as follows.

10 $$I = I_{ph} - I_{sd} \left[ {e^{{\frac{{\left( {V + IR_{s} N_{s} } \right)}}{{\left( {nN_{s} V_{t} } \right)}}}} - 1} \right] - \frac{{\left( {V + IR_{s} N_{s} } \right)}}{{R_{sh} N_{s} }}$$

## Proposed novel method

It is crucial to represent the parameter estimation as an optimization problem by adopting an objective function in order to analyze a solar system accurately. In this study, the root mean square error (RMSE), given in the following equation, is used as the objective function ($F_{Obj}$) to calculate the difference between the measured current ($I_{m}$) and estimated current ($I_{c}$) values.

11 $$F_{Obj} = \sqrt {\frac{1}{N}\mathop \sum \limits_{i = 1}^{N} \left( {I_{m} - I_{c} } \right)^{2} }$$

Here, $N$ represents the total number of data points. To estimate the current, the nonlinear equations derived from the equivalent circuits can be solved. In the conventional objective function, given a voltage measurement and a current measurement, the current is estimated using the following expression:

12 $$I_{c} = I_{ph} - I_{sd} \left[ {e^{{\frac{{\left( {V + I_{m} R_{s} } \right)}}{{\left( {nV_{t} } \right)}}}} - 1} \right] - \frac{{\left( {V + I_{m} R_{s} } \right)}}{{R_{sh} }}$$

Substituting Eq. (12) into Eq. (11), will lead to following for the SD model.

13 $$F_{Obj} = \sqrt {\frac{1}{N}\mathop \sum \limits_{i = 1}^{N} \left( {I_{m} - \left\{ {I_{ph} - I_{sd} \left[ {e^{{\frac{{\left( {V + I_{m} R_{s} } \right)}}{{\left( {nV_{t} } \right)}}}} - 1} \right] - \frac{{\left( {V + I_{m} R_{s} } \right)}}{{R_{sh} }}} \right\}} \right)^{2} }$$

For the DD model, this will be:

14 $$F_{Obj} = \sqrt {\frac{1}{N}\mathop \sum \limits_{i = 1}^{N} \left( {I_{m} - \left\{ {I_{ph} - I_{sd1} \left[ {e^{{\frac{{\left( {V + I_{m} R_{s} } \right)}}{{\left( {n_{1} V_{t} } \right)}}}} - 1\left] { - I_{sd2} } \right[e^{{\frac{{\left( {V + I_{m} R_{s} } \right)}}{{\left( {n_{2} V_{t} } \right)}}}} - 1} \right] - \frac{{\left( {V + I_{m} R_{s} } \right)}}{{R_{sh} }}} \right\}} \right)^{2} }$$

For the TD model, this will be:

15 $$F_{Obj} = \sqrt {\frac{1}{N}\mathop \sum \limits_{i = 1}^{N} \left( {I_{m} - \left\{ {I_{ph} - I_{sd1} \left( {e^{{\frac{{V + IR_{s} }}{{n_{1} V_{t} }} - 1}} } \right) - I_{sd2} \left( {e^{{\frac{{V + IR_{s} }}{{n_{2} V_{t} }} - 1}} } \right) - I_{sd3} \left( {e^{{\frac{{V + IR_{s} }}{{n_{3} V_{t} }} - 1}} } \right) - \frac{{V + IR_{s} }}{{R_{sh} }}} \right\}} \right)^{2} }$$

For the PV module model, this will be:

16 $$F_{Obj} = \sqrt {\frac{1}{N}\mathop \sum \limits_{i = 1}^{N} \left( {I_{m} - \left\{ {I_{ph} - I_{sd} \left[ {e^{{\frac{{\left( {V + IR_{s} N_{s} } \right)}}{{\left( {nN_{s} V_{t} } \right)}}}} - 1} \right] - \frac{{\left( {V + IR_{s} N_{s} } \right)}}{{R_{sh} N_{s} }}} \right\}} \right)^{2} }$$

**Figure 8:**
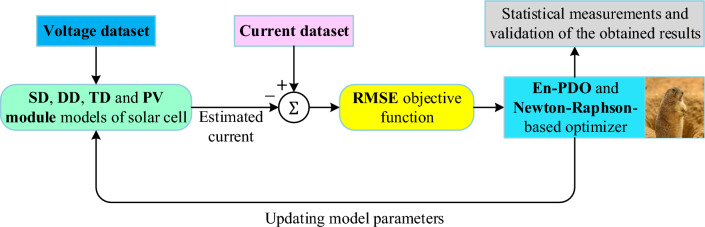
Parameter estimation process with the combination of En-PDO algorithm and Newton–Raphson technique.

Substituting ($I = I_{m}$) in Eq. (11) to estimate the current would yield inaccurate results due to the nonlinear characteristics of the models. To solve these nonlinear equations various methods can be employed, such as the Taylor series, Newton–Raphson method, Lambert W function, and others^47^. In this study, the iterative Newton–Raphson method was utilized. This method offers notable advantages, including high accuracy and relatively low computational burden. The optimization algorithm is implemented in conjunction with the Newton–Raphson method, ensuring their coordination throughout the process. Figure 8 showcases the process of parameter extraction by combining the Newton–Raphson method with the En-PDO algorithm.

The Newton–Raphson method is an iterative procedure that requires an initial point, ${x}_{0}$, and a termination condition. After $k$ iterations, the updated solution is given by $x_{k + 1} = x_{k} - f\left( x \right)/f^{\prime}\left( x \right)$. The final solution is achieved when the absolute value of $f\left( x \right)$ is less than a predefined tolerance, $\varepsilon$. For the SD and DD models, the current is computed according to Eqs. (17) and (18), respectively, by solving the nonlinear equations $f\left( x \right)$ and $g\left( x \right)$, where $x$ represents $I$.

17 $$f\left( x \right) = I_{ph} - I_{sd} \left[ {e^{{\frac{{\left( {V + xR_{s} } \right)}}{{\left( {nV_{t} } \right)}}}} - 1} \right] - \frac{{\left( {V + xR_{s} } \right)}}{{R_{sh} }} - x$$

18 $$g\left( x \right) = I_{ph} - I_{sd1} \left[ {e^{{\frac{{\left( {V + xR_{s} } \right)}}{{\left( {n_{1} V_{t} } \right)}}}} - 1\left] { - I_{sd2} } \right[e^{{\frac{{\left( {V + xR_{s} } \right)}}{{\left( {n_{2} V_{t} } \right)}}}} - 1} \right] - \frac{{\left( {V + xR_{s} } \right)}}{{R_{sh} }} - x$$

This method is utilized to compute the value of the objective function during the parameter optimization process. Throughout the optimization, the algorithm communicates the solar PV cell variables to the Newton–Raphson method, which calculates the objective function value. Specifically, the Newton–Raphson method is employed to solve the nonlinear equations in Eqs. (17) and (18) at a specific voltage, resulting in output current values with an error ($\varepsilon$) below ${10}^{-4}$. There are two significant challenges in this process. Firstly, the choice of the initial point strongly influences the final solution. Secondly, minimizing the execution time is crucial. These challenges can be overcome by a simple step. The measured current is selected as the starting point because the estimated current is expected to be close to the measured current.

## Simulation results and discussion

For the simulations of parameter extraction, we set the population size to 30 and the maximum number of iterations to 400. The algorithms were executed 30 times for each case study. We have used SD, DD and TD models of the R.T.C. France silicon solar cell along with the Photowatt-PWP201 PV model in order to demonstrate the efficacy of the proposed En-PDO for accurate extraction of related parameters.

### Simulation results of SD model

Initial evaluation of the proposed En-PDO is performed using the SD model of the commercially available and widely adopted commercial R.T.C. France silicon solar cell. The specifications of the SD model parameters employed for this study are presented in Table 4.

**Table 4:** Boundaries of parameters for SD model.

Parameter	Lower bound	Upper Bound
$I_{ph}$(A)	0	1
$I_{sd}$(µA)	0	1
$R_{s}$(Ω)	0	0.5
$R_{sh}$(Ω)	0	100
$n$	1	2

The data in Table 5 highlights the effectiveness of the En-PDO in estimating SD model parameters compared to the PDO. The En-PDO consistently produces more accurate results with smaller RMSE across all parameters. The presented data strongly supports the efficacy of the En-PDO algorithm in accurately estimating parameters. Its consistently superior performance, smaller RMSE values, and improved stability make it a compelling choice for parameter extraction tasks.

**Table 5:** Estimated parameters and statistical RMSE values for SD model with En-PDO and PDO algorithms.

Parameter	En-PDO	PDO
$I_{ph}$(A)	0.76079	0.76081
$I_{sd}$(µA)	0.31069	0.29347
$R_{s}$(Ω)	0.036547	0.036794
$R_{sh}$(Ω)	52.89	51.683
$n$	1.4773	1.4716
Minimum	**7.7299E**−**04**	7.7803E−04
Maximum	**7.7300E**−**04**	8.2141E−04
Mean	**7.7299E**−** 04**	7.9118E−04
Standard deviation	**1.8257E**−**09**	1.3260E−05

Significant values are in [bold].

**Figure 9:**
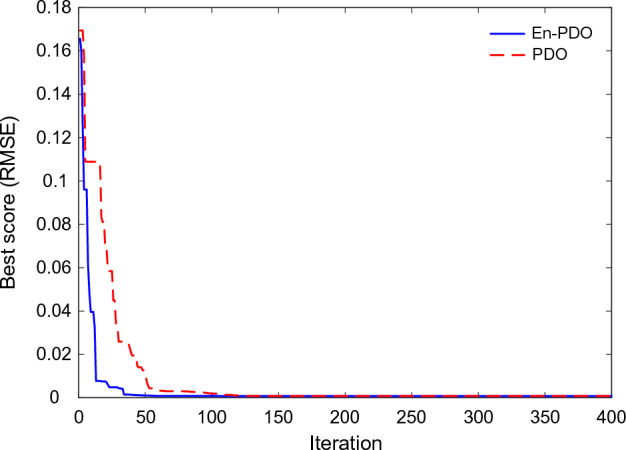
Convergence curves of En-PDO and PDO algorithms for SD model.

Figure 9 showcases the convergence behavior of the PDO and En-PDO algorithms when applied to the SD model of the commercially available R.T.C France solar cell. Examining the convergence curves, we observe that both algorithms exhibit a trend towards decreasing RMSE values. However, it is evident that the proposed En-PDO outperforms the standard PDO by consistently achieving the lowest RMSE value and attaining the best solution in earlier iterations.

**Figure 10:**
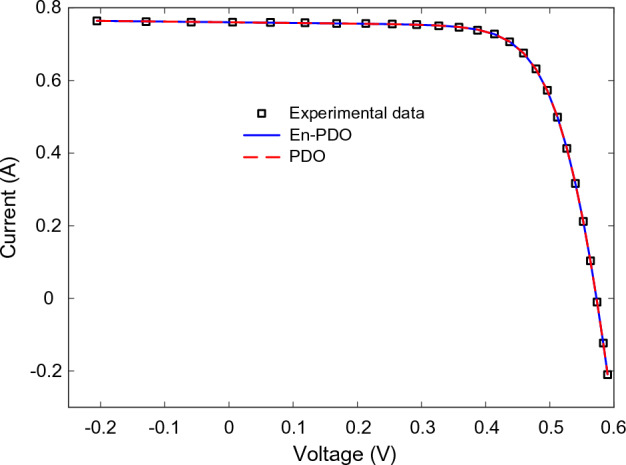
I–V curve characteristics of SD model.

**Figure 11:**
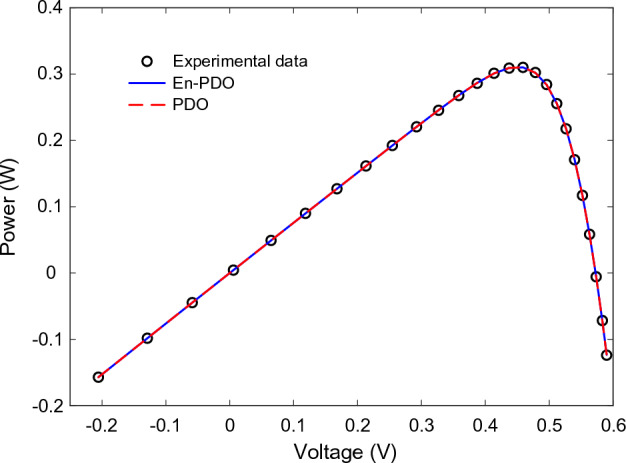
P–V curve characteristics of SD model.

Figures 10 and 11 showcase the I–V and P–V characteristics, respectively, based on the estimated parameters derived from both the PDO and En-PDO. These figures demonstrate a remarkable agreement between the estimated data and the experimental data across the entire voltage range. This close match validates the accuracy of the En-PDO in capturing the behavior of the SD model.

**Figure 12:**
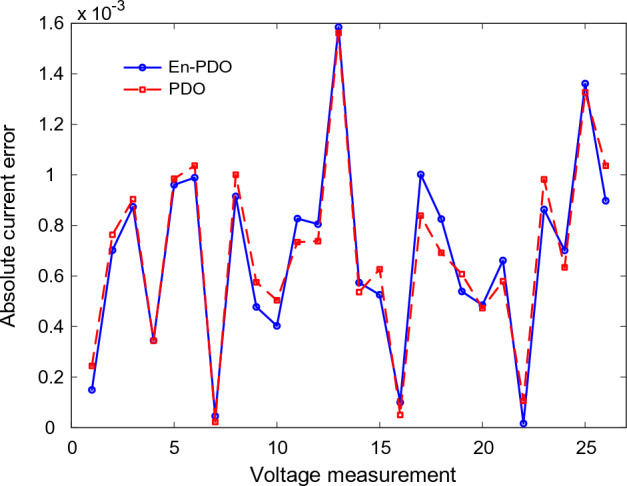
Absolute current error values for different data points in SD model.

To further emphasize the superiority of the En-PDO, Fig. 12 presents the absolute error values for different data points in the SD model. It is evident that the En-PDO consistently achieves lower error values compared to the PDO. This observation solidifies the enhanced performance of the En-PDO in accurately estimating the parameters of the SD model for the solar cell. Overall, the combination of Figs. 10, 11, and 12 provides strong evidence of the efficacy of the En-PDO for accurately modeling the SD characteristics of the R.T.C. France silicon solar cell.

### Simulation results of DD model

The evaluation of the proposed En-PDO algorithm is also performed using the DD model of the commercially available and widely adopted commercial R.T.C. France silicon solar cell. The specifications of the DD model parameters employed for this study are presented in Table 6.

**Table 6:** Boundaries of parameters for DD model.

Parameter	Lower bound	Upper bound
$I_{ph}$(A)	0	1
$I_{sd1}$(µA)	0	1
$I_{sd2}$(µA)	0	1
$R_{s}$(Ω)	0	0.5
$R_{sh}$(Ω)	0	100
$n_{1}$	1	2
$n_{2}$	1	2

The data presented in Table 7 highlights the efficacy of the En-PDO algorithm in estimating parameters compared to the PDO algorithm for the DD model. The RMSE values emphasize the superior performance of the En-PDO. The En-PDO consistently yields smaller mean RMSE values compared to the PDO suggesting better fit to the observed data and delivering more precise parameter estimates for the DD model.

**Table 7:** Estimated parameters and statistical RMSE values for DD model with En-PDO and PDO algorithms.

Parameter	En-PDO	PDO
$I_{ph}$(A)	0.7608	0.7608
$I_{sd1}$(µA)	0.094853	0.41864
$I_{sd2}$(µA)	0.99975	0.093382
$R_{s}$(Ω)	0.037538	0.037161
$R_{sh}$(Ω)	56.193	53.601
$n_{1}$	1.3851	1.6479
$n_{2}$	1.8298	1.3961
Minimum	**7.4248E–04**	7.5850E**–**04
Maximum	**7.5717E–04**	8.0828E**–**04
Mean	**7.4968E–04**	7.7130E**–**04
Standard deviation	**4.3722E–06**	1.1251E**–**05

Significant values are in [bold].

**Figure 13:**
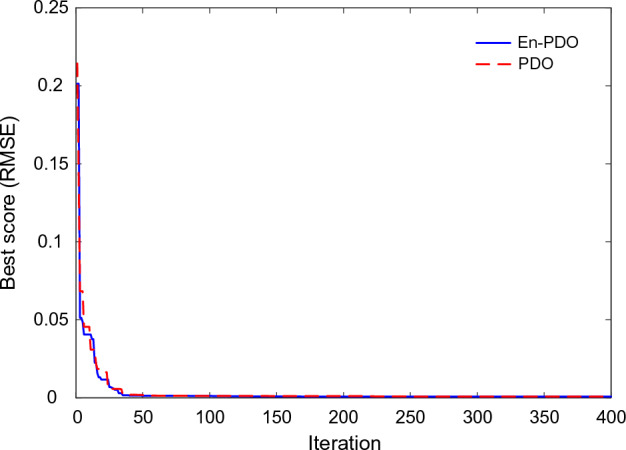
Convergence curves of En-PDO and PDO algorithms for DD model.

Figure 13 showcases the convergence behavior of the standard PDO and proposed En-PDO when applied to the DD model of the commercially available R.T.C France solar cell. Examining the convergence curves, we observe that both algorithms exhibit a trend towards decreasing RMSE values. However, it is evident that the proposed En-PDO outperforms the standard PDO by consistently achieving the lowest RMSE value and attaining the best solution.

**Figure 14:**
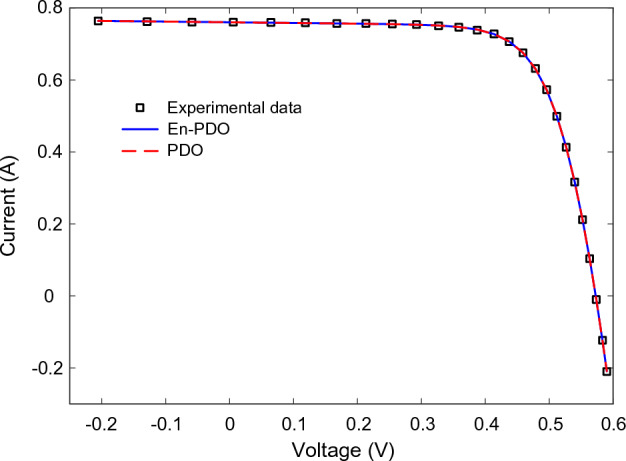
I–V curve characteristics of DD model.

**Figure 15:**
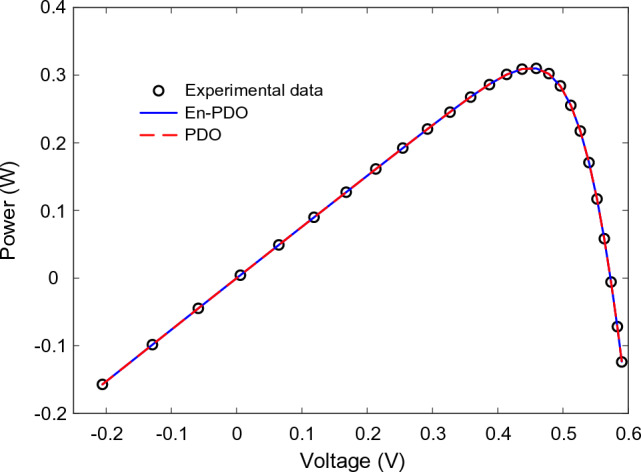
P–V curve characteristics of DD model.

**Figure 16:**
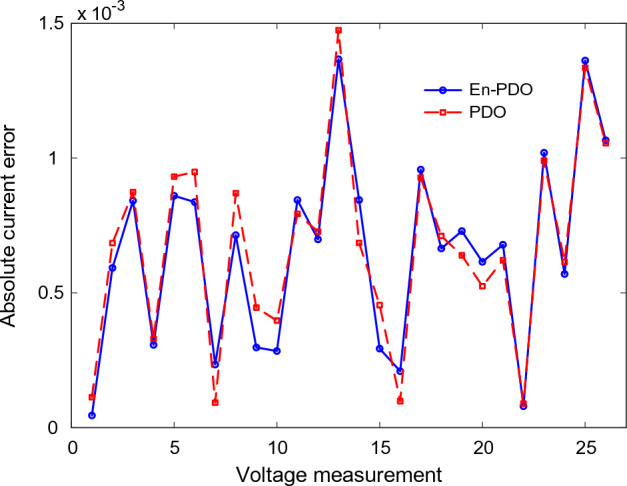
Absolute current errors values for different data points in DD model.

Figures 14 and 15 showcase the I–V and P–V characteristics, respectively, based on the estimated parameters derived from both the PDO and En-PDO. These figures demonstrate a remarkable agreement between the estimated data and the experimental data across the entire voltage range. This close match validates the accuracy of the En-PDO in capturing the behavior of the DD model. To further emphasize the superiority of the En-PDO, Fig. 16 presents the absolute error values for different data points in the DD model. It is evident that the En-PDO consistently achieves lower error values compared to the PDO. This observation solidifies the enhanced performance of the En-PDO in accurately estimating the parameters of the DD model for the solar cell.

### Simulation results of TD model

The proposed En-PDO is further assessed using the TD model of the commercially available and widely adopted commercial R.T.C. France silicon solar cell. The specifications of the TD model parameters employed for this study are presented in Table 8.

**Table 8:** Boundaries of parameters for TD model.

Parameter	Lower bound	Upper Bound
$I_{ph}$(A)	0	1
$I_{sd1}$(µA)	0	1
$I_{sd2}$(µA)	0	1
$I_{sd3}$(µA)	0	1
$R_{s}$(Ω)	0	0.5
$R_{sh}$(Ω)	0	100
$n_{1}$	1	2
$n_{2}$	1	2
$n_{3}$	1	2

The data presented in Table 9 highlights the efficacy of the En-PDO algorithm in estimating parameters compared to the PDO for the TD model. The RMSE values emphasize the superior performance of the En-PDO. The En-PDO consistently yields smaller mean RMSE values compared to the PDO suggesting better fit to the observed data and delivering more precise parameter estimates for the TD model.

**Table 9:** Estimated parameters and statistical RMSE values for TD model with En-PDO and PDO algorithms.

Parameter	En-PDO	PDO
$I_{ph}$(A)	0.7608	0.76082
$I_{sd1}$(µA)	0.81888	0.089184
$I_{sd2}$(µA)	0.66426	0.13597
$I_{sd3}$(µA)	0.13921	0.61306
$R_{s}$(Ω)	0.037467	0.037248
$R_{sh}$(Ω)	56.464	54.32
$n_{1}$	1.9997	1.7106
$n_{2}$	2	1.413
$n_{3}$	1.4105	1.8414
Minimum	**7.3832E–04**	7.4998E**–**04
Maximum	**7.5393E–04**	7.7198E**–**04
Mean	**7.4559E– 04**	7.5981E**–**04
Standard deviation	**3.8116E– 06**	7.2096E**–**06

Significant values are in [bold].

**Figure 17:**
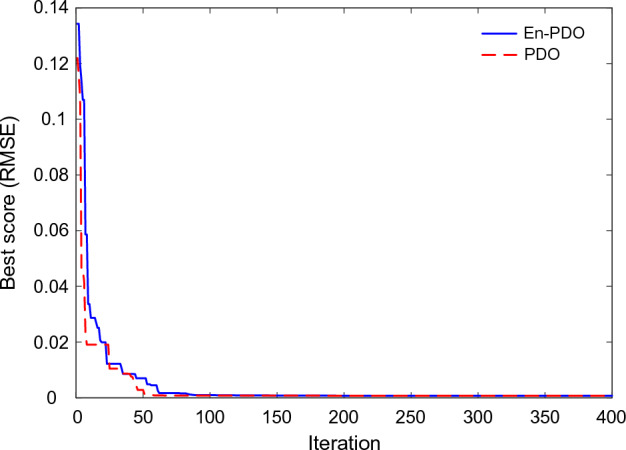
Convergence curves of En-PDO and PDO algorithms for TD model.

Figure 17 showcases the convergence behavior of the standard PDO and proposed En-PDO when applied to the TD model of the commercially available R.T.C France solar cell. Examining the convergence curves, we observe that both algorithms exhibit a trend towards decreasing RMSE values. However, it is evident that the proposed En-PDO outperforms the standard PDO by consistently achieving the lowest RMSE value and attaining the best solution in later iterations. This highlights its better performance in achieving the best solution.

**Figure 18:**
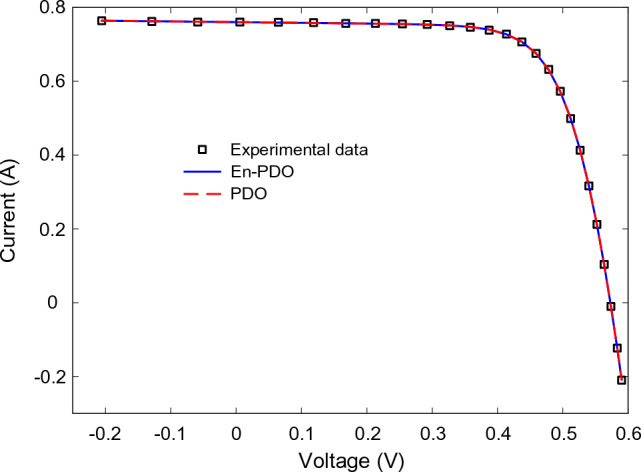
I–V curve characteristics of TD model.

**Figure 19:**
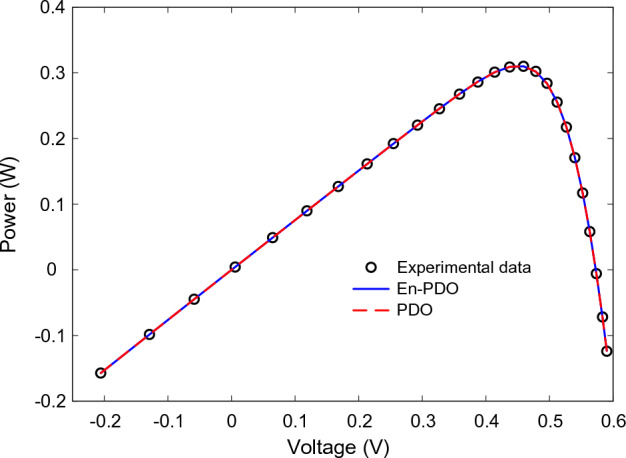
P–V curve characteristics of TD model.

**Figure 20:**
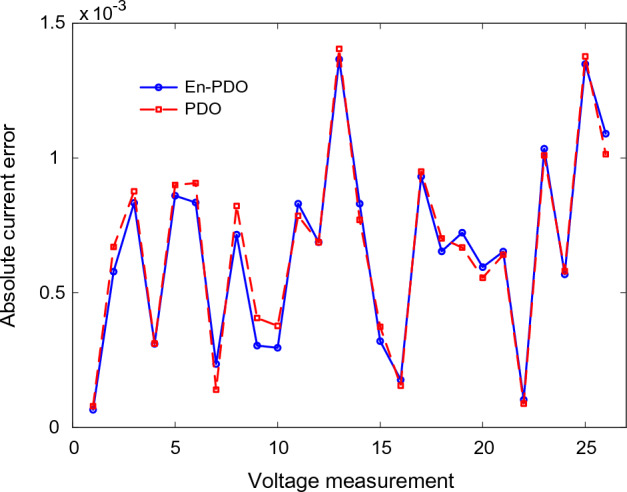
Absolute current error values for various data points in TD model.

Figures 18 and 19 showcase the I–V and P–V characteristics, respectively, based on the estimated parameters derived from both the PDO and En-PDO. These figures demonstrate a remarkable agreement between the estimated data and the experimental data across the entire voltage range. This close match validates the accuracy of the En-PDO in capturing the behavior of the TD model. To further emphasize the superiority of the En-PDO,

Figure 20 presents the absolute error values for different data points in the TD model. It is evident that the En-PDO consistently achieves lower error values compared to the PDO. This observation solidifies the enhanced performance of the En-PDO in accurately estimating the parameters of the TD model for the solar cell.

### Simulation results of PV module model

The last assessment for the proposed En-PDO is performed using the PV model of the Photowatt-PWP201 solar cell. The specifications of the PV model parameters employed for this study are presented in Table 10.

**Table 10:** Boundaries of parameters for PV module model (Photowatt-PWP201).

Parameter	Lower bound	Upper bound
${I}_{ph}$(A)	0	2
${I}_{sd}$(µA)	0	50
${R}_{s}$(Ω)	0	2
${R}_{sh}$(Ω)	0	2000
$n$	1	50

The data presented in Table 11 highlights the efficacy of the En-PDO in estimating parameters compared to the PDO for the PV model. The RMSE values emphasize the superior performance of the En-PDO. The En-PDO consistently yields smaller mean RMSE values compared to the PDO suggesting better fit to the observed data and delivering more precise parameter estimates for the PV module model.

**Table 11:** Estimated parameters and statistical RMSE values for PV module model with En-PDO and PDO algorithms.

Parameter	En-PDO	PDO
$I_{ph}$(A)	1.0314	1.0307
$I_{sd}$(µA)	2.638	2.4052
$R_{s}$(Ω)	1.2356	1.2493
$R_{sh}$(Ω)	821.61	861.29
$n$	47.598	47.256
Minimum	**2.0528E–03**	2.0882E**–**03
Maximum	**2.0528E–03**	2.1760E**–**03
Mean	**2.0528E–03**	2.1264E**–**03
Standard deviation	**6.5949E– 17**	2.3666E**–**05

Significant values are in [bold].

**Figure 21:**
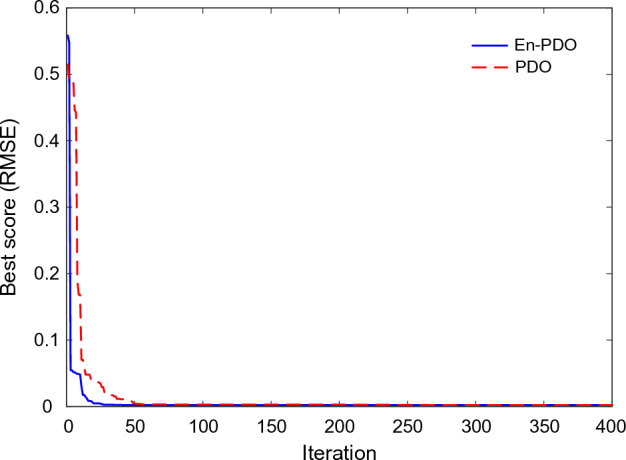
Convergence curves of En-PDO and PDO algorithms for PV module model (Photowatt-PWP201).

Figure 21 showcases the convergence behavior of the standard PDO and proposed En-PDO when applied to the PV model of the Photowatt-PWP201 solar cell. Examining the convergence curves, we observe that both algorithms exhibit a trend towards decreasing RMSE values. However, it is evident that the proposed En-PDO outperforms the standard PDO by consistently achieving the lowest RMSE value and attaining the best solution highlighting its better performance in achieving the best solution.

**Figure 22:**
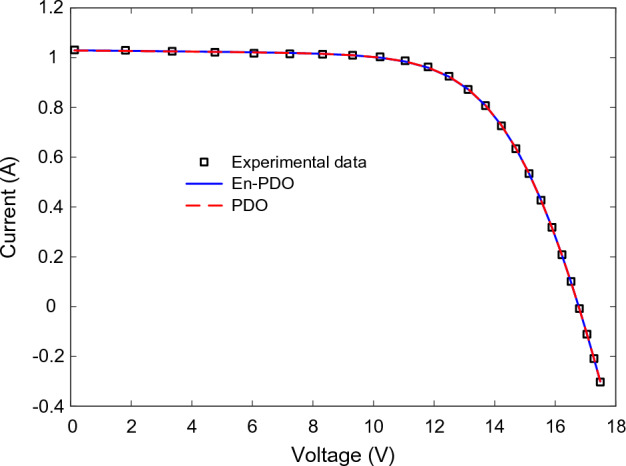
I–V curve characteristics of PV module model (Photowatt-PWP201).

**Figure 23:**
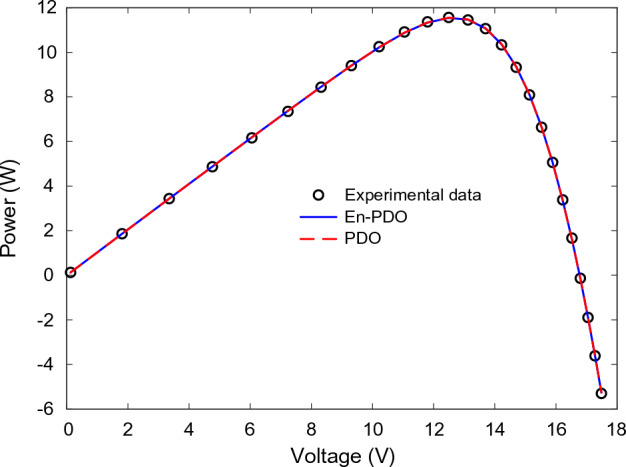
P–V curve characteristics of PV module model (Photowatt-PWP201).

**Figure 24:**
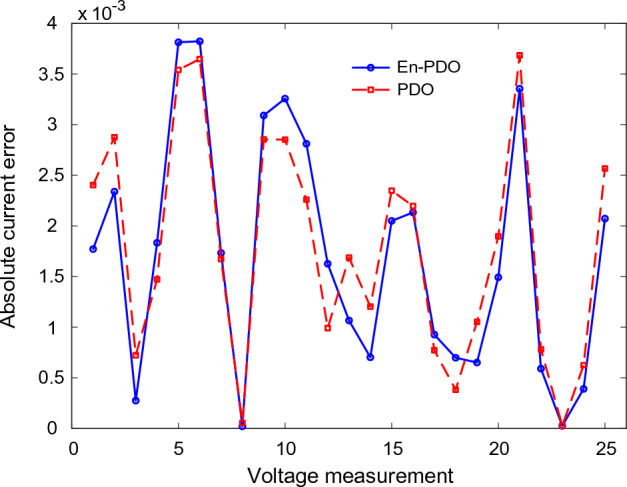
Absolute current error values for various data points in PV module model (Photowatt-PWP201).

Figures 22 and 23 showcase the I–V and P–V characteristics, respectively, based on the estimated parameters derived from both the PDO and En-PDO. These figures demonstrate a remarkable agreement between the estimated data and the experimental data across the entire voltage range. This close match validates the accuracy of the En-PDO in capturing the behavior of the PV module model. To further emphasize the superiority of the En-PDO, Fig. 24 presents the absolute error values for different data points in the PV module model. It is evident that the En-PDO consistently achieves lower error values compared to the PDO. This observation solidifies the enhanced performance of the En-PDO in accurately estimating the parameters of the PV model for the Photowatt-PWP201 solar cell.

### Comparison with recently reported algorithms

To assess the efficacy of the proposed En-PDO in the realm of photovoltaic modeling, a comparative analysis was conducted against several recently reported algorithms. The comparison includes the improved moth flame algorithm with local escape operators (IMFOL)^26^, ranking teaching–learning-based optimization (RTLBO) algorithm^27^, dynamic leader multi-verse optimizer (DLMVO)^28^ amended reptile search algorithm (OBL-RSACM)^29^, chaos game optimization-least squares (CGO-LS) algorithm^30^, artificial hummingbird optimization (AHO) algorithm^31^, elite learning adaptive differential evolution (ELADE)^32^, squirrel search algorithm (SSA)^33^, enhanced gradient-based optimizer (CCNMGBO)^34^, random reselection particle swarm optimization (PSOCS)^35^, sine cosine differential gradient based optimizer (SDGBO)^36^, differential evolution algorithm (DE)^37^, tree seed algorithm (TSA)^38^, Manta ray foraging optimization algorithm (MRFO)^39^, bald eagle search (BES) algorithm^40^, stochastic fractal search (SFS) algorithm^41^, coyote optimization algorithm (COA)^42^ and slime mould algorithm (SMA)^43^.

Table 12 provides a comprehensive comparison of the RMSE values for each algorithm across different models, including PV module model (Photowatt-PWP201) as well as SD, DD and TD models (R.T.C. France solar cell). Comparing En-PDO with other algorithms, it is evident that En-PDO consistently achieves competitive or superior performance in terms of RMSE values across all models. Notable achievements include the lowest RMSE values in the SD, DD, TD and PV models, demonstrating the efficacy of En-PDO in accurately modeling the behavior of different solar cells. The significant numerical results and the consistently superior performance of En-PDO across various models underscore its potential as an advanced optimization algorithm for photovoltaic modeling. These outcomes position En-PDO as a promising and reliable choice for optimizing parameters in the solar energy domain, showcasing its relevance and superiority compared to the array of recently reported algorithms.

**Table 12:** Comparison of RMSE values.

Algorithm	Year	R.T.C. France silicon solar cell			Photowatt-PWP201
		SD model	DD model	TD model	PV module model
En-PDO	Proposed	**7.7299E–04**	**7.4248E − 04**	**7.3832E − 04**	**2.0528E − 03**
IMFOL^26^	2023	9.8602E − 04	9.8252E − 04	Not reported	2.4252E − 03
RTLBO^27^	2023	9.8602E − 04	9.8248E–04	Not reported	2.4251E–03
DLMVO^28^	2023	9.8602E–04	9.8248E–04	Not reported	2.4251E–03
OBL-RSACM^29^	2023	9.8452E–04	9.8237E–04	Not reported	2.4251E–03
CGO-LS^30^	2023	9.8602E–04	9.8248E–04	9.8248E–04	2.4251E–03
AHO^31^	2023	7.7306E–04	9.8402E–04	Not reported	2.2953E–03
ELADE^32^	2023	9.8602E–04	9.8248E–04	Not reported	2.4251E–03
SSA^33^	2023	7.7551E–04	7.7192E–04	Not reported	Not reported
CCNMGBO^34^	2022	9.8600E–04	9.8200E–04	9.8230E–04	2.4251E–03
PSOCS^35^	2022	9.8602E–04	9.8297E–04	Not reported	2.4251E–03
SDGBO^36^	2022	9.8602E–04	9.8270E–04	9.8249E–04	2.4251E–03
DE^37^	2022	7.7692E–04	7.6300E–04	Not reported	2.0529E–03
TSA^38^	2022	9.9339E–04	9.8894E–04	Not reported	2.4326E–03
MRFO^39^	2021	7.7307E–04	7.6842E–04	7.5936E–04	Not reported
BES^40^	2021	9.8602E–04	9.8248E–04	Not reported	2.4251E–04
SFS^41^	2021	7.9310E–04	7.7827E–04	Not reported	Not reported
COA^42^	2020	7.7547E–04	7.6480E–04	7.5976E–04	2.9496E–03
SMA^43^	2020	9.8482E**–**04	9.8149E–04	9.8014E–04	2.8112E–03

Significant values are in [bold].

## Conclusion

In this study, the focus was on advancing the accuracy of PV system parameter extraction, a critical aspect of optimizing PV models. Recognizing the challenges posed by real-world operational conditions, aging effects, and the lack of instrumentation, the research underscores the significance of precise parameter identification for enhancing PV system efficiency. The primary PV models, including the SD, DD, and TD models, along with the PV module model, were investigated. The aim was to augment the accuracy of parameter identification, considering the complexities associated with diverse environmental conditions. Analytical methods, numerical operations, and metaheuristic algorithms were reviewed, with a particular focus on the limitations of existing metaheuristic algorithms. To address these limitations, the study introduced the En-PDO, a novel algorithm integrating the strengths of the PDO with RL and LSS mechanisms. The evaluation against the original PDO, coupled with a comprehensive comparison involving eighteen recent algorithms, showcased En-PDO's exceptional performance across different solar cell models and CEC2020 test functions. Application of En-PDO to SD, DD, TD, and PV module models, using standard experimental datasets and CEC2020 test functions, consistently demonstrated its superiority. The algorithm achieved competitive or superior root mean square error values, indicating its efficacy in accurately modeling the behavior of various solar cells and performing optimally on CEC functions. The key contributions of this work lie in the development and validation of En-PDO as an advanced optimization algorithm for accurate parameter estimation in solar cell models. The algorithm's innovative design, integrating nature-inspired behaviors with learning mechanisms, positions it as a robust and reliable tool for addressing the challenges of PV system parameter extraction.

Future research directions could include hybridizing En-PDO with other metaheuristic algorithms, exploring adaptability to dynamic environments, extending to multi-objective optimization, assessing scalability and parallelization capabilities, implementing in real-time applications, handling uncertainties, collaborative optimization in solar energy systems, application to emerging photovoltaic technologies, developing user-friendly interfaces, and contributing to benchmarking and standardization efforts. These avenues hold promise for advancing the field of optimization algorithms in the context of solar energy, addressing emerging challenges, and facilitating widespread adoption in both research and practical applications.

## Data Availability

The datasets used and/or analysed during the current study available from the corresponding author on reasonable request.
